# Quantitative evaluation of motion compensation in post-stroke rehabilitation training based on muscle synergy

**DOI:** 10.3389/fbioe.2024.1375277

**Published:** 2024-03-07

**Authors:** Yanhong Liu, Yaowei Li, Zan Zhang, Benyan Huo, Anqin Dong

**Affiliations:** ^1^ School of Electrical and Informatic Engineering, Zhengzhou University, Zhengzhou, China; ^2^ The Rehabilitation Department, Fifth Affiliated Hospital of Zhengzhou University, Zhengzhou, China

**Keywords:** rehabilitation training, motion compensation, surface electromyography, muscle synergy, quantitative assessment

## Abstract

**Introduction:** Stroke is the second leading cause of death globally and a primary factor contributing to disability. Unilateral limb motor impairment caused by stroke is the most common scenario. The bilateral movement pattern plays a crucial role in assisting stroke survivors on the affected side to relearn lost skills. However, motion compensation often lead to decreased coordination between the limbs on both sides. Furthermore, muscle fatigue resulting from imbalanced force exertion on both sides of the limbs can also impact the rehabilitation outcomes.

**Method:** In this study, an assessment method based on muscle synergy indicators was proposed to objectively quantify the impact of motion compensation issues on rehabilitation outcomes. Muscle synergy describes the body’s neuromuscular control mechanism, representing the coordinated activation of multiple muscles during movement. 8 post-stroke hemiplegia patients and 8 healthy subjects participated in this study. During hand-cycling tasks with different resistance levels, surface electromyography signals were synchronously collected from these participants before and after fatigue. Additionally, a simulated compensation experiment was set up for healthy participants to mimic various hemiparetic states observed in patients.

**Results and discussion:** Synergy symmetry and synergy fusion were chosen as potential indicators for assessing motion compensation. The experimental results indicate significant differences in synergy symmetry and fusion levels between the healthy control group and the patient group (*p* ≤ 0.05), as well as between the healthy control group and the compensation group. Moreover, the analysis across different resistance levels showed no significant variations in the assessed indicators (*p* > 0.05), suggesting the utility of synergy symmetry and fusion indicators for the quantitative evaluation of compensation behaviors. Although muscle fatigue did not significantly alter the symmetry and fusion levels of bilateral synergies (*p* > 0.05), it did reduce the synergy repeatability across adjacent movement cycles, compromising movement stability and hindering patient recovery. Based on synergy symmetry and fusion indicators, the degree of bilateral motion compensation in patients can be quantitatively assessed, providing personalized recommendations for rehabilitation training and enhancing its effectiveness.

## 1 Introduction

The Global Burden of Disease (GBD) research data revealed that in 2019, there were over 100 million stroke cases worldwide (Feigin and Stark, 2021). The number of patients in China is as high as 17.8 million (Tu and Wang, 2023). Stroke incidents often result in impairment of the motor cortex and its descending spinal pathways, causing functional limitations in limb movements. Statistics indicate that roughly 80% of stroke survivors experience upper limb motor dysfunction with unilateral limb motor impairment caused by stroke being the most common (Mazzoleni et al., 2018), making rehabilitation crucial for restoring lost functionality (Gauthier et al., 2008; Huang et al., 2022; Xie et al., 2023). Rehabilitation techniques encompass professional therapeutic interventions, the use of rehabilitative exoskeleton robots (Kim et al., 2012; Louie et al., 2020; Singh et al., 2021; Nolan et al., 2023) and active rehabilitation devices (Sugihra et al., 2018). However, the scarcity of rehabilitation physicians and the cumbersome nature of rehabilitative exoskeleton robots hinder their widespread use.

Active rehabilitation devices involve patients utilizing their less affected limb to assist in rehabilitation exercises, such as using hand-crank devices (Kraaijenbrink et al., 2021) and Bobath hand techniques (Pathak et al., 2021). This method promotes the initiative of patients during the training process. Studies have suggested that simultaneous training of both limbs provides additional stimulation to the brain, aiding in rehabilitation (Woldag et al., 2004; Renner et al., 2005). Nonetheless, this training method raises concerns about motion compensation. The affected limb’s reduced function leads to continuous reliance on the unaffected limb. Additionally, Calabro and Perez (2016) indicated that simultaneous action on both sides would adversely affect the movement of the affected side by the healthier side, as evidenced by comparisons of several movement indicators. The continuous exertion by the healthier side easily leads to muscle fatigue, while the affected side, due to its functional deficit, is prone to fatigue as well. This imbalanced force exertion on both sides of the limbs caused by compensation can significantly hinder a patient’s recovery.

Therefore, timely rehabilitation assessment of rehabilitation training can effectively reduce the impact of motion compensation and muscle fatigue. Presently, upper limb rehabilitation assessment methods, including Brunnstrom Recovery Stage (Meng et al., 2022), Fugl-Meyer Assessment (DJ, 2002), and Modified Ashworth Scale (Ansari et al., 2012), possess comprehensive evaluation criteria. They heavily rely on clinical expertise and possess subjectivity. Li et al. (2022) integrated surface electromyography (sEMG) signals and motion information for the quantitative assessment of hand function. However, these methods face challenges in assessing motion compensation. Therefore, timely rehabilitation assessment of rehabilitation training can effectively reduce the impact of motion compensation and muscle fatigue on rehabilitation training. Timely detection of motion compensation remains challenging in clinical practice, delaying patient recovery and potentially resulting in permanent functional deficits in the affected limb.

The muscle synergy theory describes the inherent neuro-muscular control mechanism in the human body, suggesting that motor neurons do not solely control individual muscles but recruit multiple muscles simultaneously to execute coordinated movements (Aoi and Funato, 2016; Hirashima and Oya, 2016; Zhao et al., 2019). Tang et al. (2014) applied Pearson correlation analysis on muscle synergy among different healthy subjects executing similar tasks revealed a correlation coefficient of up to 0.85. Chen et al. (2023) identified shared and specific synergies in six upper limb actions, forming a basis for the muscle synergy theory. Brambilla and Scano (2022) utilized experimental and simulated data to investigate the influence of the number of muscles on the structure and quantity of synergies. Their conclusions suggest that both a low and high number of muscles can yield relatively high similarity in synergy. Additionally, a lower number of muscles might potentially underestimate the dimensionality of motor control, thereby potentially providing a basis for motor control. Pan et al. (2021) further analyzed that different combinations of the original muscle synergies could achieve complex movements in different planes. The distinct topology of muscle synergy networks among different tasks demonstrates the significant differences, thus affirming the potential of the muscle synergy theory in understanding human motor control mechanisms and their impact on neurorehabilitation. Due to the interpretability of muscle synergy in human movement mechanisms, this theory is often employed for patients’ rehabilitation assessments.

Commonly used approaches for applying muscle synergy to rehabilitation assessment involve comparing muscle synergies between healthy individuals and patients to gauge changes in patient synergy indicators. In experiments conducted by Funato et al. (2022), both healthy individuals and stroke patients were tasked with executing the 37-item tasks from the Fugl-Meyer assessment method. The corresponding muscle synergies were analyzed to explore the relationship between synergy characteristics and stroke-related motor impairments. Ultimately, it was deduced that muscle synergy serves as an effective method in stroke assessment. Ma et al. (2021) extracted upper limb muscle synergies from healthy subjects and stroke patients, analyzing the inherent consistency of the patients’ multiple experimental outcomes. This analysis revealed lower inherent consistency in stroke patients compared to healthy subjects, accompanied by a higher level of synergy complexity. Sheng et al. (2022) introduced a novel assessment method known as the Muscle Synergy Space (MSS) model, aimed at evaluating post-stroke motor function. By comparing muscle synergy characteristics between healthy individuals and stroke patients, the model’s effectiveness was demonstrated, providing scientific guidance for rehabilitation.

Another approach considers that patients often exhibit better functionality in their unaffected (healthy) side. Therefore, by comparing and analyzing the muscle synergy between the unaffected and affected sides, it is also feasible to assess the motor function of the affected side. This primarily involves an analysis from the perspective of the correlation and integration level of muscle synergy between both sides of the body (Cheung et al., 2012; Pan et al., 2018). However, while the first method uses healthy subjects’ synergy as a reference to reflect patient synergy defects, it fails to assess the balance of bilateral muscle coordination during coordinated movements. The second method involves experiments solely focusing on independent movements on both sides, reflecting only some indicators of changes during independent movements. Consequently, the results do not adequately indicate differences in muscle coordination levels due to compensation by the unaffected side.

The innovation of this study lies in not only analyzing the patient’s own muscle synergy indicators but also conducting a significance analysis between the results of patients and healthy subjects. This evaluation is based on indicators such as the symmetry and fusion degree of muscle synergy on both sides of the body, aiming to assess the issue of motion compensation. Collecting surface electromyography signals from patients and healthy participants during bilateral movement, will facilitate analysis of indicators variations. This will quantify patients motion compensation through comparative analysis based on muscle synergy indicators. Considering that resistance is often applied during experimental procedures to enhance rehabilitation training, and patients tend to experience muscle fatigue, an analysis of muscle synergy indicators has been conducted under various resistance levels and fatigue statuses. The main contributions of this paper can be summarized as follows.

1. The combined muscle synergy extraction method utilizing Principal Component Analysis (PCA) and Non-negative Matrix Factorization (NMF) ensures the stability of muscle synergy patterns and subsequent analytical results.
2. Designing experimental paradigms sensibly, using synergy symmetry and synergy fusion indicators, validated the feasibility of quantifying motion compensation issues through muscle synergy.
3. By comparing data between the healthy group and the patient group, as well as between the healthy group and the simulated group, the impact of resistance level and fatigue status on motion compensation is analyzed using significance level indicators.

The rest of paper is organized as follows. Section 2 describes the recruited subjects, experimental protocols, and muscle synergy quantification indicators. Section 3 presents the analysis results of the experiments, while Section 4 delves into the discussion of these analytical findings. Conclusion are set out in Section 5.

## 2 Materials and methods

This study primarily analyzes the coordination level between the two sides of the human body from the perspective of muscle synergy. The muscle synergy extraction algorithm was employed to extract muscle synergies and activation coefficient matrices from sEMG signals. Muscle synergy reflects the recruitment pattern of muscles within a muscle group and can also serve as a measure of muscle symmetry, while activation coefficients indicate the degree of involvement of each muscle synergy. The symmetry and fusion indicators of bilateral muscle synergy reflect the coordination of muscles, and the effectiveness of applying this method to detect motion compensation can be determined through a significant difference analysis between healthy subjects and patients.

### 2.1 Subjects

Eight stroke patients (S1-S8, mean age 45 ± 15 years) and eight healthy subjects (H1-H8, mean age 24 ± 2 years) participated in this experiment. All patients were capable of independently completing a minimum of 20 min of hand-cranked rehabilitation training. All eight healthy subjects were right-handed. The information of stroke patient is available in Table 1. The experiment was in accordance with the declaration of Helsinki and received approval from the Fifth Affiliated Hospital of Zhengzhou University. All subjects provided informed consent before participating in the experiment.

**TABLE 1:** Stroke subjects.

Subject	Age	Affected side	Stroke type	Brunnstrom
S1	16	*R*	*Ischemic*	3
S2	60	*R*	*Ischemic*	3
S3	58	*R*	*Ischemic*	4
S4	32	*L*	*Ischemic*	5
S5	37	*R*	*Ischemic*	5
S6	58	*L*	*Ischemic*	3
S7	55	*L*	*Ischemic*	4
S8	45	*L*	*Ischemic*	5

### 2.2 Experiment protocols

#### 2.2.1 Experimental platform

**FIGURE 1:**
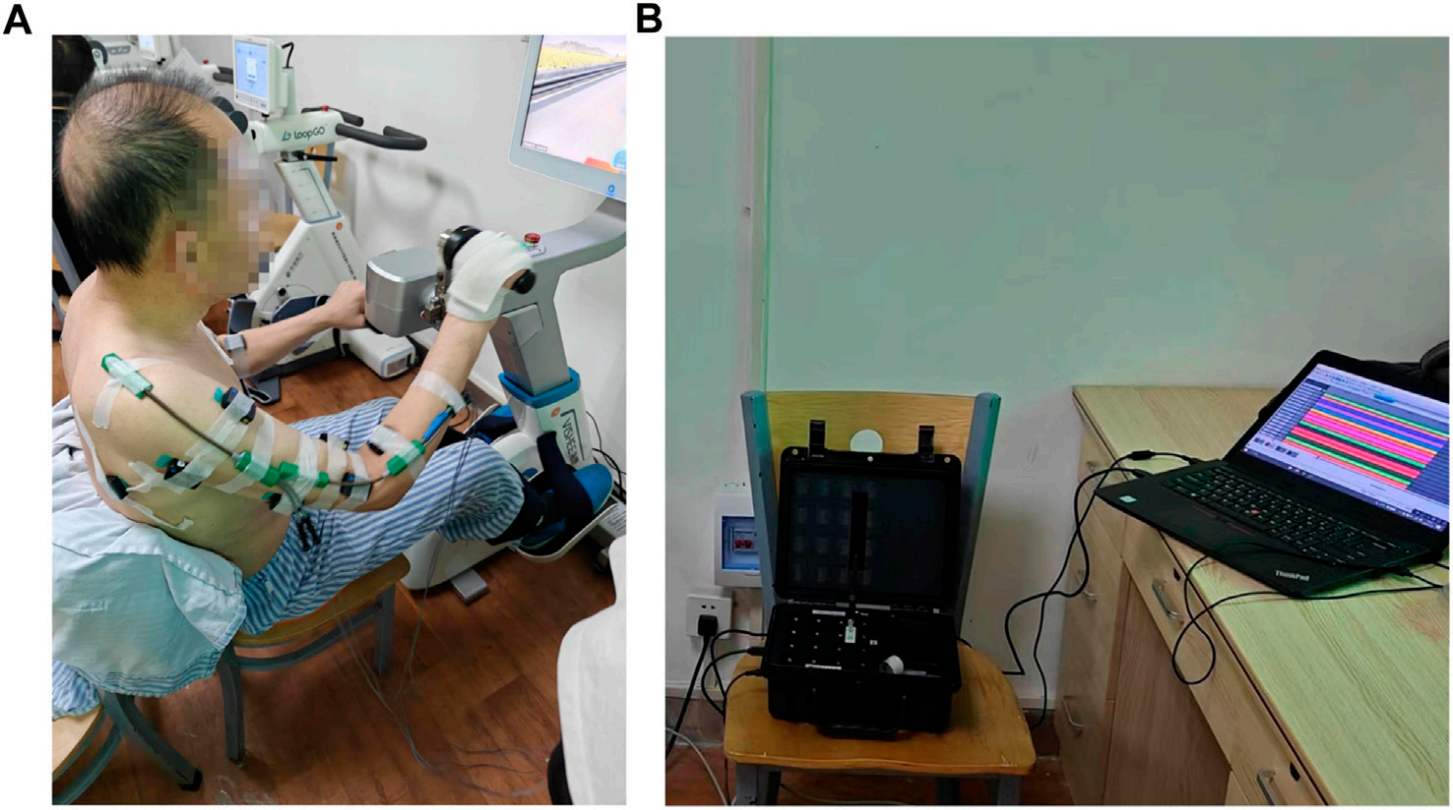
**(A)** Experimental setup and equipment-hand-cycling. The device allows for adjustable training resistance. **(B)** Experimental data acquisition equipment-Delsys. The device can simultaneously collect sEMG signals from up to 16 muscles.

**FIGURE 2:**
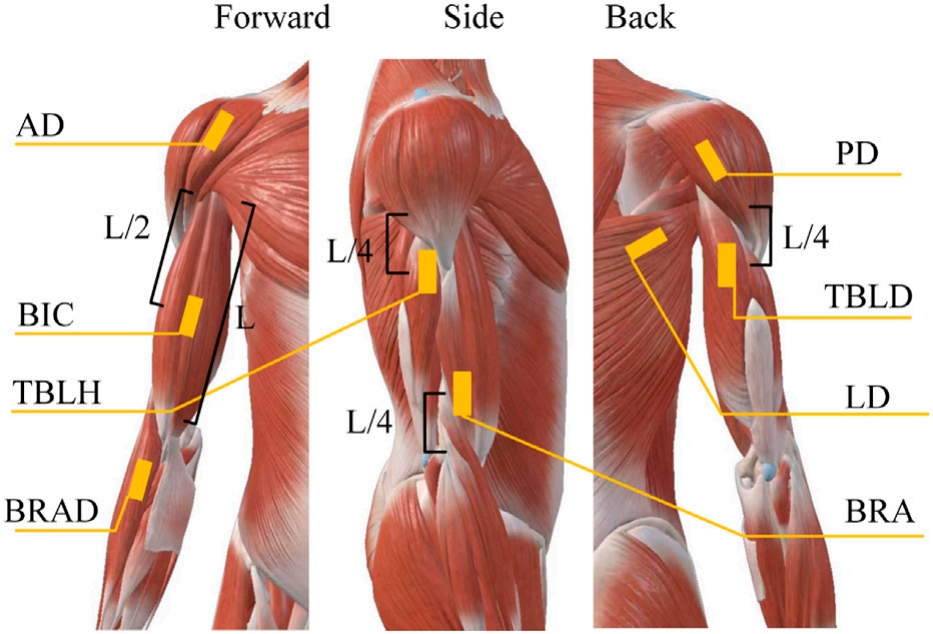
Electrode positions for sEMG signals acquisition. The positions of each muscle were predetermined through referencing anatomical charts.

This study utilized a coordinated bilateral hand-cycling as the experimental apparatus, as shown in Figure 1A, and employed the multi-channel wireless sEMG signals sensor Delsys for data acquisition in Figure 1B.The sEMG sampling rate was 1,926 Hz. Data collection was performed on eight muscle groups on both the left and right sides of the human body, with sensor attachment positions on the right side illustrated in Figure 2, which were mirrored symmetrically on the left side.

The muscles associated with rehabilitation training for hand-operated carts are the Biceps Brachii (BIC), Brachialis (BRA), Anterior Deltoid (AD), Brachioradialis (BRAD), Posterior Deltoid (PD), Triceps Brachii Long Head (TBLD), Triceps Brachii Lateral Head (TBLH), and Latissimus Dorsi (LD), as determined through examination of anatomical charts and experimental analysis. After wiping the skin with alcohol wipes, affixing the sensor to the designated location, and subsequently securing it more firmly with medical tape, this approach aims to diminish noise caused by skin perspiration and artifacts from sensor movement.

#### 2.2.2 Experimental paradigm

**FIGURE 3:**
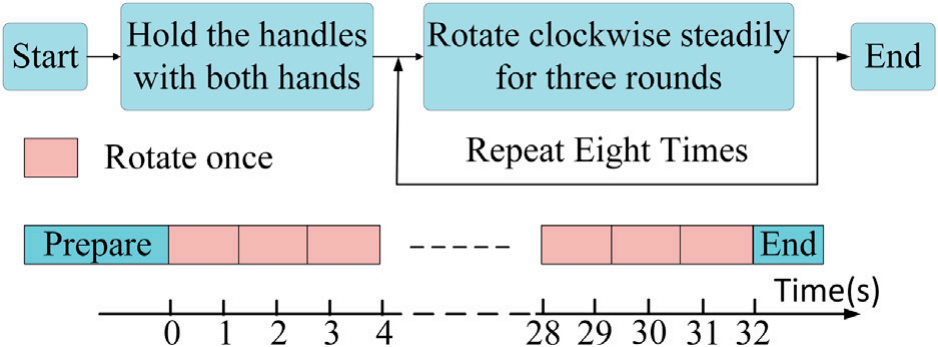
Experimental paradigm. Every 3 complete rotational cycles constitute a single large cycle, and at least 8 large cycles (32 s) were collected as one dataset.

The overall experimental paradigm is indicated in Figure 3. The participants were initially briefed on the experimental procedure, where three rotation cycles were considered a complete co-contraction extraction cycle. Most patients completed three full rotation cycles within a 4 s interval, considering this timeframe as a major cycle for coordinated data extraction. Healthy subjects followed a similar pace, leading to a total data collection time of 32 s. To prevent any interference from the initiation and cessation movements at the start and the end, a minimum data collection duration of 36 s was ensured for each participant, with intermittent rest periods. Two distinguishable resistance levels were set, labeled as Resistance level 1 (R 1) and Resistance level 2 (R 2). Once data collection for both resistance levels was completed, patients continued with at least 20 min of rehabilitation training. Subsequently, data were gathered at the R 1 to capture fatigue data.

All healthy subjects underwent the following simulated compensation experiment based on the original experiment, with the resistance set at R 1.

Case 1: Maintain a balanced movement on both sides as much as possible.

Case 2: Sustain stable force exertion on the right side, with occasional engagement of the left side in movement, simulating a milder degree of motion compensation.

Case 3: The left side exerts no force and is entirely driven by the right side, simulating a more severe form of motion compensation.

### 2.3 The preprocessing of sEMG

The preprocessing of sEMG signals involved several steps. First, a Butterworth high-pass filter with a cutoff frequency of 40 Hz was applied to the acquired sEMG signals (Chen et al., 2023). Next, a 50 Hz notch filter was used to remove powerline interference. The signals were mean-centered and rectified. Subsequently, Finite Impulse Response (FIR) low-pass filter with a cutoff frequency of 20 Hz was applied to extract the signal envelope (Pan et al., 2018). Root mean square smoothing was employed to further refine the signals, eliminating the aberrant electrical noise and ensuring a smoother envelope. Finally, a normalization process was carried out to ensure that the contribution of smaller muscle groups was adequately represented.

**FIGURE 4:**
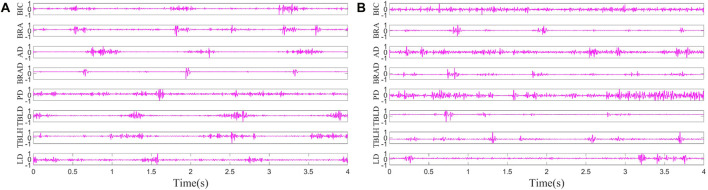
**(A)** sEMG of H1. **(B)** sEMG of S1.

The pre-processing results of sEMG signals from the left side of H1 in Case 1 and the affected side of S1 are shown in Figure 4. The result involves the normalization of sEMG, constraining its range to −1 to 1, and the envelope signals have not yet been extracted. It is evident that the periodicity of data from healthy subjects is more intuitively apparent.

### 2.4 The extraction of muscle synergies

When controlling limb movements, the human body doesn’t individually control each muscle, rather it coordinates the entire muscle group through the spinal cord. Muscle synergy can be extracted from preprocessed sEMG signals using feature extraction algorithms, as described in Eq. 1

$$V^{m\times n}=W^{m\times r}H^{r\times n}+E,$$ (1)

where *V* is the preprocessed surface sEMG signals, with *m* as the number of sampling channels and *n* as the number of sampling points, *W* is the muscle synergy matrix, with *r* is the number of muscle synergies, *H* is the activation coefficient matrix, *E* is the obtained residual error. The extraction of muscle synergies involves two steps.

Step 1: PCA is employed to obtain the feature matrix and principal component matrix. The selection is made for feature vectors and their corresponding principal components equal to the number of muscle synergies.

Step 2: The results obtained in the previous step are taken in absolute values as the initial values for NMF (Lee and Seung, 1999).

The number of muscle synergies is determined by the error between the decomposition results and the original signals. This error is represented by the variance accounted for (*VAF*) (Cheung et al., 2012), as shown in Eq. 2

$$VAF=1-\frac{\|V-V^{\prime }{\|}_{2}}{\|V{\|}_{2}},$$ (2)

where *V* is the preprocessed surface sEMG signals matrix, *V*′ is the reconstruction matrix. When the *VAF* is excessively high, it fails to achieve effective dimensionality reduction, as redundant information cannot be completely eliminated. Conversely, if the *VAF* is too low, it may result in the loss of valuable information. Therefore, the minimum number of synergies is chosen when *VAF* exceeds 80%.

### 2.5 Synergy symmetry and fusion

In the study, muscle synergies were extracted using the mentioned method from both sides of both healthy subjects and patients. The symmetry of muscle synergy refers to the correlation calculation results of the extracted muscle synergies on both sides of the human body. This indicator can reflect the balance of movement on both sides, thereby indicating the degree of motion compensation. Although the muscle synergy modules were relatively stable, the order in which muscle synergies were extracted exhibited randomness. To establish a more meaningful order of muscle synergies, the muscle synergy order within the synergy matrix was rearranged to optimize the overall synergy correlation. This was done by calculating synergy correlations between the extracted muscle synergies on both sides. The correlation calculation method employed in the study was the Pearson correlation coefficient (Lalumiere et al., 2022), as shown in Eq. 3

$$\rho =\frac{\sum \left(X-\bar{X}\right)\left(Y-\bar{Y}\right)}{\sqrt{\sum {\left(X-\bar{X}\right)}^{2}\sum {\left(Y-\bar{Y}\right)}^{2}}},$$ (3)

where *X* and *Y* correspond to two synergy vectors that require correlation determination, and $\bar{X}$ and $\bar{Y}$ represent the respective means. Synergy vector refers to the results of each column in the extracted synergy matrix. In this study, the number of channels in the multi-channel signals is 8, and the number of elements in each synergy vector is also 8.

In cases where one side displays reduced functionality, resulting in abnormal muscle synergy, a phenomenon known as synergy fusion may manifest. The fusion indicator of muscle synergy denotes that the muscle synergy on one side of the subject is formed by the fusion of muscle synergies from the other side. This indicator reflects the variation in the force exerted by individual muscles on both sides during the coordinated movement process in patients, thereby indicating the degree of motion compensation. Specifically, when muscle synergy on the affected side becomes aberrant while that on the unaffected side remains relatively normal, a fusion of synergies may occur as in Eq. 4. In this study, the calculation of synergy fusion on the affected side is carried out utilizing the least squares method (Cheung et al., 2012).

$$W_{i}^{a}=\overset{N^{u}}{\underset{k=1}{\sum }}m_{k}^{i}W_{k}^{u},\;i=1\;\ldots N^{a},$$ (4)

where $W_{i}^{a}$ represents the *i* − *th* muscle synergy on the affected side, *N* ^*a*^ signifies the number of synergies on the affected side, $W_{k}^{u}$ denotes the *k* − *th* muscle synergy on the healthy side, fusion coefficient $m_{k}^{i}$ denotes the contribution level of the *k*th healthy side muscle synergy, and *N* ^*u*^ represents the number of muscle synergies on the healthy side. When the fusion coefficient exceeds 0.2, it indicates the involvement of one synergy in composing the synergy on the other side. If only one fusion coefficient exceeds 0.2, the presence of fusion is not considered. If more than two fusion coefficients exceed 0.2, it is considered that another synergy exists in fusion on the opposite side.

### 2.6 Significance analysis

This study conducted experiments with two resistance levels and under fatigue status for both healthy subjects and patients. The determination of significance levels for computed indicators relating to muscle synergy under various conditions involves employing distinct statistical methodologies contingent upon the distribution of the data. Typically, in instances where the data adheres to a normal distribution, significance analysis is carried out using one-way analysis of variance (ANOVA). The Lilliefors test was utilized to assess the normal distribution of characteristics associated with muscle synergy (Abdi and Molin, 2007). This method is deemed appropriate for small sample sizes, and if the data deviates from a normal distribution (*p* ≤ 0.05), the Kruskal–Wallis test is subsequently applied to assess the significance of differences (Bala et al., 2023).

## 3 Results

### 3.1 The number of muscle synergies

**FIGURE 5:**
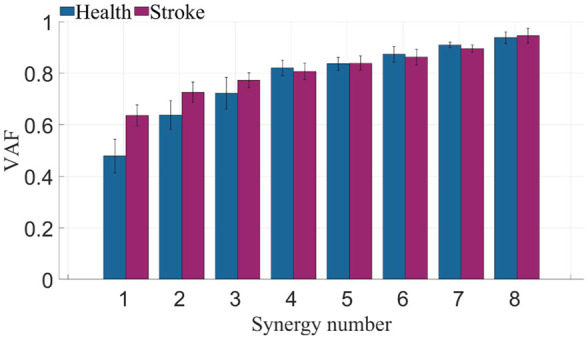
*VAF* of healthy subjects and stroke patients. The blue bars represent the *VAF* across different muscle synergy numbers for healthy subjects, while the purple bars represent the *VAF* across different muscle synergy numbers for patients.

Following preprocessing of multi-channel sEMG from both sides of all participants, a combination of PCA and NMF algorithms was employed to determine different counts of co-activation, aiming to extract muscle synergies. The *VAF* were computed under various co-activation counts. As depicted in Figure 5, muscle synergies on the left side of healthy subjects and the affected side of patients exhibited *VAF* values exceeding 0.8 when the synergies count exceeded 4. Consequently, The number of muscle synergies extracted for both healthy subjects and patients was determined to be 4. This synergy number adequately reconstructs the information of the original data while eliminating some redundant information.

### 3.2 Symmetry of bilateral synergy

**FIGURE 6:**
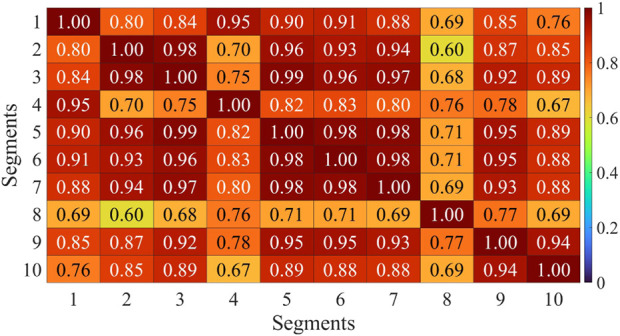
Correlation analysis of muscle synergy with equidistant offset of H1. Horizontal axis and vertical axis corresponds to the sequential numbers used for muscle synergy extraction data sets.

**FIGURE 7:**
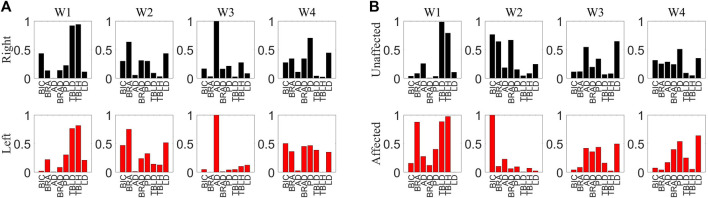
**(A)** Muscle synergy of H1. The upper section representing the synergy of the right side muscles and the lower section depicting the synergy of the left side muscles. **(B)** Muscle synergy of S1. The upper section representing the synergy of the healthy side muscles and the lower section depicting the synergy of the affected side muscles.

Muscle synergies were extracted from 8 channels of sEMG signals on both sides, and the Pearson correlation coefficient was employed to assess the symmetry between bilateral muscle synergies. This study involved the extraction and analysis of sEMG signals during simultaneous movement of the left and right sides. Considering the potential impact of phase deviations between both sides, an initial computation involved evaluating the correlation of muscle synergy extraction results for ten segments with a phase deviation of 200 sampling points. As depicted in Figure 6, the majority of data segments exhibited a symmetry above 0.85, indicating minimal variations in synergy with slight time deviations. Extract muscle synergy from both sides of healthy participants and patients, as illustrated in Figure 7.

**FIGURE 8:**
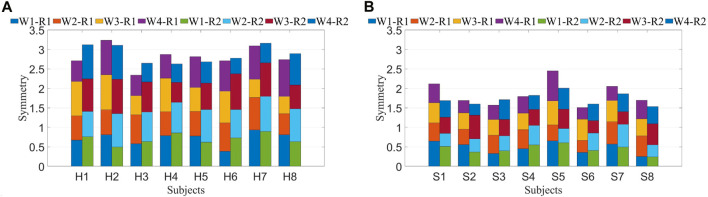
**(A)** Symmetry of synergy in healthy subjects. **(B)** Symmetry of synergy in stroke patients. Each bar consists of four segments, where each segment represents symmetry between both sides for a synergy. W1-W4 denote four synergies, while R 1 and R 2 represent two resistance levels.

To minimize the errors introduced by periodic variations, the average of the symmetry results from eight data segments was calculated to represent the final data, aiming to reduce the impact of small temporal deviations on muscle synergy analysis. The symmetry of muscle synergies for eight patients is shown in Figure 8. Each column consists of 4 segments, representing the symmetry values of each synergy. The overall synergy symmetry for healthy subjects is mostly above 2.5, while for patients, it is predominantly below 2.5.

**FIGURE 9:**
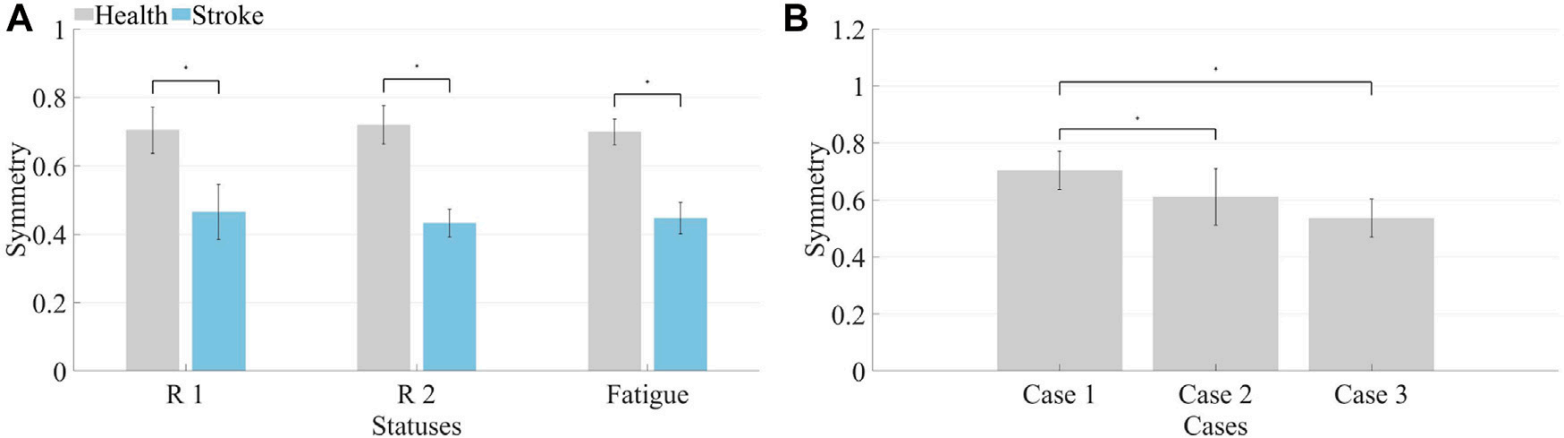
**(A)** Synergy symmetry in different states. The left bars indicate the average symmetry between both sides in three statuses for healthy subjects, while the right bars represent the average symmetry across three conditions for patients. **(B)** Synergy symmetry in simulated compensation cases. Calculate the average synergy symmetry for each case across different healthy subjects.

Similar computations performed on muscle synergies in fatigue status yielded identical outcomes. Under different statuses, the Pearson correlation coefficient was utilized to compute the symmetry of muscle synergies across all healthy subjects and patients, generating mean and standard deviation values. After conducting the Lilliefors test for normality on the data and determining that it does not follow a normal distribution, the Kruskal–Wallis test was applied to determine if there were significant differences in symmetry between R 1 and R 2 for both healthy subjects and patients. Similar computations were conducted for pre-fatigue and post-fatigue conditions, as depicted in Figure 9A.

The symmetry in healthy subjects under R 1 (0.70 ± 0.07) and R 2 (0.72 ± 0.06) did not show significant differences (*p* > 0.05). Additionally, the symmetry before fatigue under R 1 did not significantly differ from the symmetry after fatigue (0.69 ± 0.04, *p* > 0.05). For patients, symmetry under R 1 (0.46 ± 0.08) and R 2 (0.43 ± 0.04) showed no significant differences (*p* > 0.05), and the symmetry before fatigue under R 1 did not significantly differ from the symmetry after fatigue (0.44 ± 0.05, *p* > 0.05). However, healthy subjects exhibited significantly higher synergy symmetry compared to patients (*p* ≤ 0.05). Consequently, it is inferred that resistance levels and fatigue statuses do not significantly impact bilateral muscle synergy symmetry in the human body. However, significant differences exist in the symmetry of muscle synergies between patients and healthy subjects.

The average and standard deviation of muscle synergy symmetry across different subjects under the three cases were computed which is shown in Figure 9B. For most subjects, the synergy symmetry was highest in Case 1, lowest in Case 3, and the significance level was determined. The average symmetry for Case 2 was (0.61 ± 0.10), and for Case 3, it was (0.54 ± 0.07). The symmetry in Case 1 was significantly higher than that of Case 3 (*p* ≤ 0.05).

### 3.3 Fusion of bilateral synergy

This study computes the fusion of muscle synergies, considering that all healthy subjects are right-handed. For healthy subjects, the fusion of left-side muscle synergies was calculated from the right-side muscle synergies. For patients, the fusion of affected-side muscle synergies was derived from the unaffected-side muscle synergies.

**FIGURE 10:**
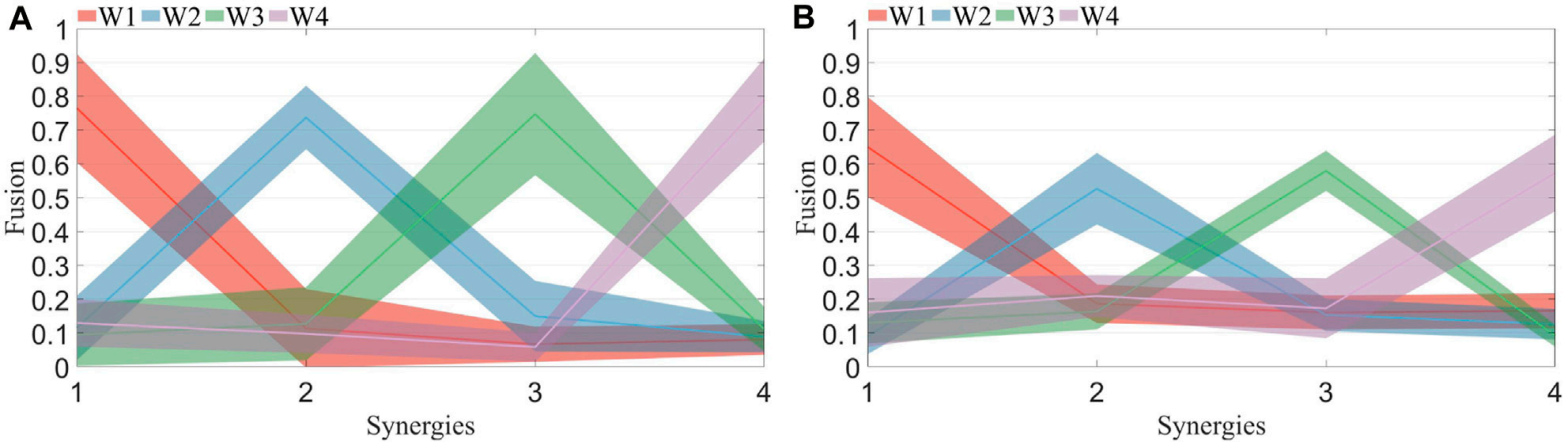
**(A)** Synergy fusion in healthy subjects. Each color corresponds to the four points on the x-axis, representing the fusion of the four synergies from the left side to the right side. The solid line indicates the mean, while the shaded area represents the standard deviation. **(B)** Synergy fusion in patients. Each color corresponds to the four points on the x-axis, representing the fusion of the four synergies from the healthy side to the affected side. The solid line indicates the mean, while the shaded area represents the standard deviation.

The fusion levels of muscle synergies were calculated for both healthy subjects and patients under R 1, R 2, and fatigue statuses. H1 represents healthy subject 1. The computed fusion levels among healthy subjects is shown in Figure 10A. The solid line represents the average across all subjects, while the shaded area indicates the standard deviation. Each curve corresponds to four points, representing the reconstruction coefficients of that synergy by four synergies from the other side. A coefficient exceeding 0.2 suggests the involvement of the corresponding synergy in the reconstruction process. When the number of reconstructed synergies exceeds 2, fusion of that synergy is considered to be present. From the graph, it is evident that most curves display a single prominent peak, with other points mostly below 0.2. The synergy fusion status among patients under R 1 is shown in Figure 10B. Each curve not only exhibits a dominant peak but also contains additional points with values exceeding 0.2. This suggests the presence of multiple synergies formed through fusion of synergies from the unaffected side.

**FIGURE 11:**
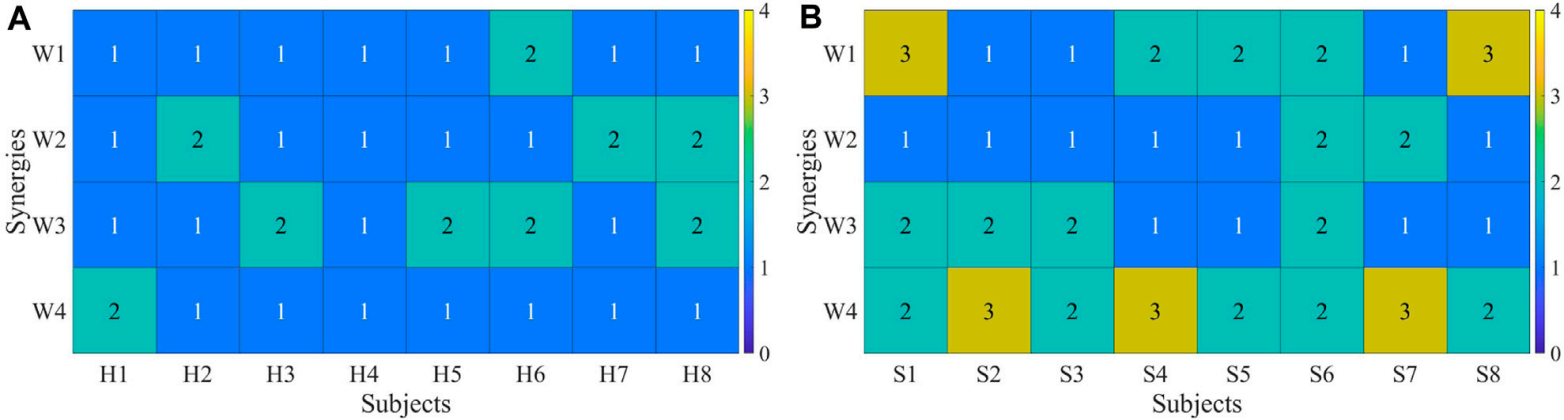
**(A)** Synergy fusion in healthy subjects. **(B)** Synergy fusion in patients. Each column represents a healthy subject **(A)** or a patient **(B)**, and each row depicts the degree of synergy fusion. A value above 2 indicates the presence of fusion.

Each participant’s synergy fusion status was plotted individually. The calculation of synergy fusion among healthy subjects under R 1 is shown in Figure 11A. Each column represents a participant, while the values in each row denote the fusion status of that synergy. Values exceeding 2 indicate the presence of fusion for a given synergy. Most healthy participants exhibit fusion in only one out of the four muscle synergies, except for participants 6 and 8. The calculation of synergy fusion among patients under R 1 is shown in Figure 11B. Healthy subjects typically show fusion in two or fewer muscle synergies, while patients often exhibit fusion in more than two synergies, with some demonstrating fusion in all four synergies.

**FIGURE 12:**
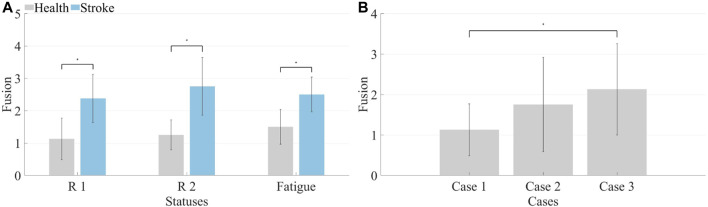
**(A)** Synergy fusion in different states. The left side represents healthy subjects, while the right side represents patients. **(B)** Synergy fusion in simulated compensation status. Calculate the average synergy fusion count for each case across different healthy subjects.

Both healthy subjects and patients underwent analysis for synergy fusion levels under R 1, R 2, and fatigue statuses, as illustrated in Tables 2, 3. The numbers in the table represent the count of synergistic fusion occurrences among the four muscle synergies for each subject. The average and standard deviation of fused synergies were calculated for healthy subjects and patients, as depicted in Figure 12A. Subsequently, the significance level of differences among various statuses was determined. The outcomes indicate an absence of significant differences in synergy fusion levels between the two resistance levels and pre-fatigue and post-fatigue statuses. The number of fusions for healthy participants under R 1 (1.13 ± 0.64) and R 2 (1.25 ± 0.46) showed no significant difference (*p* > 0.05). Also, there was no significant difference between the pre-fatigue and post-fatigue fusion counts under R 1 (1.5 ± 0.53) among healthy participants (*p* > 0.05). For patients, there was no significant difference in the fusion counts between R 1 (2.37 ± 0.74) and R 2 (2.75 ± 0.89, *p* > 0.05). Similarly, there was no significant difference between pre-fatigue and post-fatigue fusion numbers under R 1 (2.5 ± 0.53) among patients (*p* > 0.05). However, the number of fusions in healthy participants was significantly lower than in patients (*p* ≤ 0.05).

**TABLE 2:** Fusion of healthy subjects.

Subject	H1	H2	H3	H4	H5	H6	H7	H8
R 1	1	1	1	0	1	2	1	2
R 2	1	1	1	1	1	2	1	2
Fatigue	2	1	2	1	1	2	1	2
Case 1	1	1	1	0	1	2	1	2
Case 2	0	1	3	1	3	1	3	2
Case 3	1	2	3	2	3	0	3	3

**TABLE 3:** Fusion of stroke subjects.

Subject	S1	S2	S3	S4	S5	S6	S7	S8
R 1	3	2	2	2	2	4	2	2
R 2	2	4	3	2	2	3	4	2
Fatigue	2	3	3	3	2	4	3	2

It is illustrated that the computation of synergy fusion levels among healthy subjects in simulated compensation scenarios in Table 2. The average results for multiple healthy subjects are depicted in Figure 12B. The results indicate a gradual increase in synergy fusion levels from Case 1 to Case 3. The average fusion count for Case 2 is (1.75 ± 1.17), and for Case 3 is (2.13 ± 1.23). The fusion count in Case 1 is significantly lower than in Case 3 (*p* ≤ 0.05).

### 3.4 Influence of muscle fatigue on synergy

**FIGURE 13:**
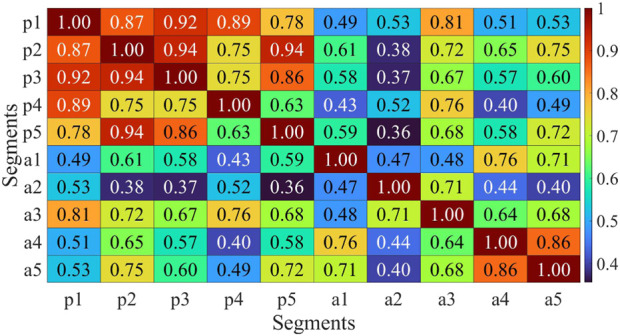
Multicycle synergy correlation before and after fatigue of H2. p1-p5 represent the five consecutive cycle data before fatigue, while a1-a5 represent the five consecutive cycle data after fatigue.

The analysis conducted above indicates that muscle fatigue does not significantly impact the symmetry of coordination and fusion levels during bilateral coordinated movements in the human body. However, an assessment was performed on the correlation of muscle synergies pre and post fatigue. Muscle synergies were extracted from five movement cycles before and after fatigue for each participant, and the correlation between these synergies was computed pairwise. It is presented that the computed results for one of the patients, demonstrating a reduction in synergy correlation post-fatigue in Figure 13. Moreover, a slight decline in correlation was observed in the five sets of post-fatigue muscle synergy data, which was consistent across multiple participants. This suggests that muscle fatigue leads to an increase in the instability of muscle synergies, which could impede effective rehabilitation training and assessment. Therefore, mitigating the occurrence of muscle fatigue should be a priority to ensure optimal conditions for rehabilitation training and evaluation.

## 4 Discussion

The present study takes into account the compensation phenomenon that occurs during bilateral auxiliary movement. Compensation is the phenomenon of leveraging the function of muscles on both sides due to functional deficiencies. In the process of bilateral auxiliary rehabilitation training, patients tend to excessively rely on the unaffected side due to the functional impairment of the affected side. Current rehabilitation assessment methods often rely heavily on clinical expertise, such as FMA and Fugl-Meyer scales. However, these methods lack the ability to promptly detect the occurrence of compensation behaviors in patients. Prolonged reliance on the unaffected side may lead to muscle atrophy and permanent loss of function on the affected side.

To quantify compensation behaviors on both sides, this study designed experiments using a handcart and incorporated simulated compensation experiments. Healthy subjects’ right hand was designated as the unaffected side, while the left side was either completely restrained or occasionally engaged in the handcart’s rotational movement. Motion compensation manifests in the coordination of muscle exertion levels. Given that sEMG reflect the degree of muscle exertion in each muscle, bilateral balance handcart experiments were conducted to collect sEMG signals from corresponding areas on both sides of healthy subjects and patients. According to muscle synergy theory, human body movement is achieved through the linear combination of multiple synergy modules. Extracting muscle synergy from multi-channel sEMG signals is essential. Presently, NMF is primarily used for muscle synergy extraction, mainly due to the interpretability of its non-negative results. However, NMF’s drawback lies in its multiple solutions, indicating instability as it might yield different outcomes when run multiple times on the same dataset. This variability significantly impacts the application of muscle synergy in rehabilitation assessment, leading to substantial differences between multiple analytical results. Some studies have employed PCA to extract muscle synergy. PCA’s advantage lies in its capability to extract stable muscle synergies. However, its results often contain negative values, limiting its application in rehabilitation assessment. Nevertheless, there are studies integrating muscle synergies extracted by PCA into rehabilitation exoskeletons’ dimensionality reduction control (Alibeji et al., 2015).

This study analyzes the symmetry of muscle synergy on both sides of the body, demanding high stability in extracted synergies. To obtain stable muscle synergies, sEMG signals underwent filtering and smoothing, followed by normalization for each channel to prevent smaller muscles’ participation from being overshadowed by larger muscle groups. Considering that NMF algorithm results are significantly influenced by the initial input matrix, this study employed PCA for preliminary decomposition of multi-channel surface electromyographic signals on each side. The absolute value of the decomposed results was then processed and utilized as the input matrix for the NMF algorithm, resulting in relatively stable muscle synergy outcomes. Symmetry and fusion levels of muscle synergy extracted from both sides of the body were primarily analyzed. Furthermore, variations in muscle synergy indicators during compensation simulated experiments were examined to identify potential indices applicable for quantifying compensation behavior.

### 4.1 The number of muscle synergy

The determination of the quantity of muscle synergies is not automatically established through an algorithm but involves selecting various synergy numbers, computing the *VAF* values, and assessing them through plotting. The computed *VAF* values for synergy extraction on the affected side of patients and the left side of healthy subjects is shown in Figure 5. Typically, a threshold is set to determine the number of synergies. Considering the redundancy in sEMG, this study set the threshold at 0.8, which reflects 80% of the original data’s information in the obtained synergy results. From the graph, notable differences in *VAF* between patients and healthy subjects are observed when the synergy numbers are small. However, when the synergy numbers exceed 4, most participants in both patient and healthy subject groups exhibit a *VAF* greater than 0.8. Similar results are obtained when analyzing the other side. Consequently, the quantity of muscle synergies was established as 4.

### 4.2 The symmetry of bilateral muscle synergy

The primary consideration was the phase shift in bilateral movement during the handcart training, which, reflected in sEMG signals, represents a time shift. Approximately 1.3 s are required for a full rotation, resulting in a time offset of roughly half a rotation between the two sides. With a sampling rate of 1,926 Hz, this offset corresponds to 1,251 data points. Therefore, this study employed 8,000 as a data window, ensuring the inclusion of at least three complete rotation cycles, encompassing the initiation, termination, and transitional phases of the movement in the sEMG signals. Muscle selection was performed at intervals of 200 from 10 segments of sEMG signals, followed by muscle synergy extraction.

The assessment of muscle synergy variations across different signal segments, as shown in Figure 6, indicated that the correlation of most muscle synergies exceeded 0.85, suggesting minimal phase shifts and negligible changes in synergy. To minimize errors induced by shifts, the study computed muscle synergy symmetry for eight data segments and averaged them to yield the final results. The extracted results of muscle synergies for healthy subjects and patients are shown in Figure 7. The right panel displays the muscle synergies on both sides for patients. Compared to healthy subjects, there are notable differences in muscle synergies on both sides for patients.

The initial phase involved the calculation of muscle synergy symmetry between both sides of the participants, as depicted in Figure 8. Each bar consists of four segments, representing the symmetry of individual muscle synergies. The bar chart visually illustrates a significant difference in overall synergy symmetry between healthy subjects and patients. The overall synergy symmetry for healthy subjects is mostly above 2.5, whereas for patients, it tends to be below 2.5. The analysis revealed an average synergy symmetry of 0.70 for healthy subjects, contrasting with only 0.46 for patients, as depicted in Figure 9A. Notably, synergy symmetry in healthy subjects was significantly higher than in patients. Analyzing the simulated compensation muscle synergy states of healthy subjects, as illustrated in Figure 9B. The synergy symmetry demonstrated a declining trend across the three cases, with Case 1 exhibiting significantly higher symmetry than Case 2 and Case 3 (*p* ≤ 0.05). This stepped pattern across the three cases indicates that muscle synergy symmetry could quantify compensation behaviors. A significant analysis of muscle synergy results under different resistance levels (*p* > 0.05) suggests no significant differences in symmetry outcomes under varying resistance levels. Geng et al. (2020) also suggests high stability in muscle synergy analysis under different resistance Level. Furthermore, a significant analysis of pre- and post-fatigue muscle synergy results revealed (*p* > 0.05), indicating that muscle fatigue does not significantly impact synergy symmetry.

### 4.3 The fusion of bilateral muscle synergy

The fusion of synergies implies when the muscle synergies on the affected side are composed of contributions from more than two healthy-side muscle synergies. The fusion coefficients were computed using non-negative least squares. Results for healthy subjects are illustrated in Figure 10A, while those for patients are depicted in Figure 10B. A comparison reveals that most maximum fusion coefficients in patients are smaller than those in healthy subjects, whereas most minimum fusion coefficients are larger in patients than in healthy subjects. Subsequently, the fusion status of synergies was computed and plotted in Figure 11, where each column of four numbers corresponds to the fusion status of four synergies for a participant. Values above 2 indicate the presence of fusion in that synergy. The analysis indicates that the synergy fusion counts in healthy subjects are mostly below 2, whereas patients tend to have a synergy fusion count significantly above 2. The average synergy counts among different resistance levels and post-fatigue in various participants was calculated, followed by significant analysis depicted in Figure 12A. The synergy fusion counts in patients is significantly higher than in healthy subjects. After fatigue, there is a slight increase in fusion counts for healthy subjects, which, however, is not significant (*p* > 0.05). Patients show the maximum fusion synergy count after increased resistance levels, but neither resistance levels nor fatigue resulted in significant changes. Regarding the calculation of synergy fusion under simulated compensation cases in Figure 12B, a stair-like increment is observed across the three cases. Notably, the fusion count in Case 1 is significantly lower than in Case 3 (*p* ≤ 0.05). Furthermore, the fusion count in Case 3 is more similar to that of the patients, indicating that fusion indicators can quantify compensation evaluation on both sides. The calculation of different cycle synergy correlations pre-and post-fatigue among participants revealed a decrease in synergy correlations for most subjects after fatigue, indicating increased instability in human control due to muscle fatigue in Figure 13. This factor might not favor rehabilitation training and assessment. Thus, efforts should be made to avoid muscle fatigue during rehabilitation training.

## 5 Conclusion

This study proposed a muscle synergy-based assessment method to objectively quantify motion compensation in post-stroke hemiplegia patients. The results demonstrated that synergy symmetry and synergy fusion indicators effectively assessed motion compensation during hand-cycling tasks. Patients with poorer limb functionality exhibited lower synergy symmetry, indicating decreased coordination. Simulated compensation experiments of healthy subjects further validated these results. Moreover, neither resistance levels nor fatigue status demonstrates a significant impact on these indicators. However, fatigue led to reduced stability in motor control for both patients and healthy subjects. The study emphasizes the importance of minimizing muscle fatigue during rehabilitation training. Overall, synergy-based indicators provide a quantitative assessment of bilateral motion compensation, offering personalized recommendations for effective rehabilitation.

## Data Availability

The raw data supporting the conclusion of this article will be made available by the authors, without undue reservation.
